# Identification of peptide domains involved in the subcellular localization of the feline coronavirus 3b protein

**DOI:** 10.1099/jgv.0.001321

**Published:** 2019-09-04

**Authors:** Delphine D. Acar, Veerle J. E. Stroobants, Herman Favoreel, Xavier Saelens, Hans J. Nauwynck

**Affiliations:** ^1^​ Department of Virology, Parasitology and Immunology, Faculty of Veterinary Medicine, Ghent University, Merelbeke, Belgium; ^2^​ VIB-UGent Center for Medical Biotechnology, VIB, Ghent, Belgium; Department of Biochemistry and Microbiology, Ghent University, Ghent, Belgium

**Keywords:** feline coronavirus, accessory protein, nucleolus, mitochondria

## Abstract

Feline coronavirus (FCoV) has been identified as the aetiological agent of feline infectious peritonitis (FIP), a highly fatal systemic disease in cats. FCoV open reading frame 3 (ORF3) encodes accessory proteins 3a, 3b and 3 c. The FCoV 3b accessory protein consists of 72 amino acid residues and localizes to nucleoli and mitochondria. The present work focused on peptide domains within FCoV 3b that drive its intracellular trafficking. Transfection of different cell types with FCoV 3b fused to enhanced green fluorescent protein (EGFP) or 3×FLAG confirmed localization of FCoV 3b in the mitochondria and nucleoli. Using serial truncated mutants, we showed that nucleolar accumulation is controlled by a joint nucleolar and nuclear localization signal (NoLS/NLS) in which the identified overlapping pat4 motifs (residues 53–57) play a critical role. Mutational analysis also revealed that mitochondrial translocation is mediated by N-terminal residues 10–35, in which a Tom20 recognition motif (residues 13–17) and two other overlapping hexamers (residues 24–30) associated with mitochondrial targeting were identified. In addition, a second Tom20 recognition motif was identified further downstream (residues 61–65), although the mitochondrial translocation evoked by these residues seemed less efficient as a diffuse cytoplasmic distribution was also observed. Assessing the spatiotemporal distribution of FCoV 3b did not provide convincing evidence of dynamic shuttling behaviour between the nucleoli and the mitochondria.

## Introduction

Coronavirus infections have emerged in various species of mammals and birds and are generally associated with a wide spectrum of respiratory, intestinal and systemic infections. Feline coronaviruses (FCoV) can cause both enteric and systemic diseases in domestic and wild *Felidae* and occur as two pathotypes for which the disease-causing potential is determined by their cell tropism. The feline enteric coronavirus (FECV) is an enzootic enteropathogenic virus that replicates in intestinal epithelial cells after oral uptake [1–5]. Most infections with FECV are subclinical and rarely give rise to symptoms such as mild diarrhoea or reduced appetite [5]. The highly virulent feline infectious peritonitis virus (FIPV) arises within an individual cat by mutations in the FECV genome, which enables efficient viral replication in monocytes and macrophages [6–9]. FIPV spreads systemically and causes the highly fatal disease known as feline infectious peritonitis virus (FIP), which is characterized by a fibrinous and granulomatous serositis, fibrinous effusions in the affected body cavities and/or multifocal granulomatous vascular lesions in several organs [10–12]. Besides these two pathotypes, two FCoV serotypes can be distinguished. Serotype I FCoVs are most prevalent in the field [13–18], but difficult to grow *in vitro*. To date, the only cell line that allows sustainable growth of serotype I FCoV strains is a feline intestinal epithelial cell (fIEC) line established by Desmarets and colleagues [19]. The less prevalent serotype II FCoV arose by double recombination between serotype I FCoV and canine coronavirus (CCoV), resulting in a FCoV with spike and open reading frame 3 (ORF3) sequences of CCoV origin [20, 21].

The FCoV genome consists of a single-stranded positive-sense RNA strand of about 29 kb that codes for 16 non-structural, 4 structural and 5 accessory proteins. ORF3 encodes accessory proteins 3a, 3b and 3 c. The ORF3-encoded proteins do not seem to be essential for sustainable growth *in vitro* in *Felis catus* whole foetus cells and feline blood monocytes [22, 23], although virus titres were markedly lower after the inoculation of feline blood monocytes with a FIPV ORF3 deletion mutant compared to the wild-type [22]. Moreover, inoculation of cats with recombinant FIPV lacking ORF3 failed to induce typical FIP clinical signs. This loss of virulence *in vivo* suggests a potential role of the ORF3-encoded proteins in virus–host interactions [23]. The FCoV 3b amino acid sequence comprises about 72 amino acid residues and is well conserved within pathotypes [24]. It was recently reported that 3b localizes to mitochondria and nucleoli, both in the presence and absence of other viral proteins [25]. However, the function(s) of this accessory protein remain unknown. Interestingly, the accessory 3b protein of severe acute respiratory syndrome coronavirus (SARS-CoV), which comprises 154 amino acids, also translocates to mitochondria and nucleoli [26–28], even though it does not share any amino acid sequence homology with FCoV 3b. SARS-CoV 3b induces apoptosis [29, 30], necrosis [29] and cell-cycle arrest [30], and suppresses type I IFN expression induced by retinoic acid-induced gene 1 and the mitochondrial antiviral signalling protein (MAVS) [26].

Many viral proteins are known to target the nucleolus and may interfere with cellular stress responses, cell cycle regulation and apoptotic processes. In addition, these proteins can modify cellular and viral transcription or recruit specific nucleolar components, such as nucleolin, to facilitate viral replication (reviewed in [31–34]). Upon synthesis in the cytoplasm, nucleolar proteins first migrate to the nucleus and then subsequently to the nucleolus. Small soluble proteins can passively diffuse from the cytoplasm through the nuclear pore complex into the nucleoplasm (reviewed in [35–37]). Transport of larger proteins to the nucleus, however, is an ATP-dependent active process driven by nuclear localization signals (NLSs), which are typically characterized by clusters of basic amino acids (reviewed in [38–42]). These NLSs are recognized by importins that mediate transport into the nucleus. Similarly, shuttling of proteins from the nucleus back to the cytoplasm is established by nuclear export signals (NESs) that are recognized by exportins such as CRM1. Nucleolar import of proteins can be achieved through association with nucleolar components such as rRNA or nucleolar proteins [32, 34, 43, 44]. For various proteins, a nucleolar localization signal (NoLS) was identified that targets these proteins to the nucleoli. However, NoLSs are not well characterized and no consensus motif is available. In general, they display a high content of basic residues and thus resemble or incorporate NLSs (reviewed in [32–34, 44, 45]).

Viral proteins that target mitochondria have been implicated in Ca^2+^ homeostasis, energy production and apoptosis. Furthermore, such proteins can cause oxidative stress and modulate the mitochondrial membrane permeability and potential, and antiviral responses that emanate from a mitochondria-associated complex such as MAVS. Some of these viral proteins mimic or hijack mitochondrial proteins to their advantage, or alter the dynamics or intracellular localization of mitochondria (reviewed in [46–51]). Most mitochondrial proteins are encoded by nuclear genes, synthesized in the cytosol and subsequently imported into mitochondria. This process is typically driven by an N-terminal cleavable presequence, the mitochondrial targeting peptide (mTP), which interacts with the translocase of the outer membrane (TOM) complex, more specifically with the Tom20 and Tom22 proteins in the outer mitochondrial membrane (reviewed in [51, 52]). Even though a consensus sequence for these mTPs has not been established, there are common features with respect to amino acid composition and physicochemical properties. For example, mitochondrial targeting peptides are often rich in positively charged residues, such as arginine, almost always lack negatively charged residues and generally have the ability to form amphipathic α-helices with hydrophobic residues on one side and positively charged residues on the other [53–58]. The hydrophobic surface is recognized by Tom20 and the positively charged surface interacts with Tom22 [59–61]. Upon import into the mitochondrial matrix, the N-terminal presequence is cleaved off by the mitochondrial processing peptidase (MPP) and the protein subsequently translocates to one of the submitochondrial compartments [51].

In this study, we investigated the subcellular localization of FCoV 3b and characterized the molecular determinants that drive intracellular targeting of this accessory protein.

## Methods

### Plasmid construction: pEGFP–3b, p3b–EGFP, p3×FLAG–3b, p3b–3×FLAG

The FCoV genome was amplified from viral RNA present in faeces from cats that had been experimentally infected with FECV UCD [62] using the primers listed in Table 1. The faeces were collected 9 days post-infection and the complete viral genome sequence was obtained (GenBank accession number KU215423). Viral RNA was extracted with the QIAamp Viral RNA Mini kit (Qiagen) according to the manufacturer’s instructions and cDNA was produced with the SuperScript III First-Strand Synthesis System (Invitrogen). Next, the 3b open reading frame was amplified by PCR with Herculase II Fusion DNA Polymerase (Agilent Technologies). To create p3b–EGFP, where 3b is fused at its C-terminus with enhanced green fluorescent protein (EGFP), the forward primer contained an *Eco*RI site, a Kozak sequence (GCCGCCACC) and the 5′ end of the 3b gene. The reverse primer contained a *Bam*HI site and the 3′ end of the 3b gene, but no 3b stop codon to allow read-through transcription (Table 1). The restriction enzyme-digested PCR product was cloned into pcDNA3.1(−)-EGFP with a mutated EGFP start codon (TTG instead of ATG). To construct pEGFP–3b, where 3b is fused at its N-terminus with EGFP, the forward primer comprised an *Eco*RI site and the 5′ end of the 3b gene with a mutated 3b start codon (TTG instead of ATG), and the reverse primer contained a *Bam*HI site and the 3′ end of the 3b gene (Table 1). The PCR product was cloned into pcDNA3.1(−)-EGFP in which the stop codon of EGFP was deleted. Similarly, p3b–3×FLAG and p3×FLAG–3b constructs were created using pcDNA3.1(−)-3×FLAG. The correctness of the cloned expression constructs was confirmed by Sanger sequencing.

**Table 1.** Primers used for the construction of the wild-type and truncated 3b

Construct name	Mutation	Polarity	Sequence
pEGFP–3b and p3×FLAG–3b	Wild-type	Sense^*a*,*b*^ Antisense^*a*^	5′ TCTGCA**GAATTC** *TTG*CCAAACTTCAGCT 3′ 5′ CCCCTT**GGATCC**TCATTTTCGCGCTGCGTTTAGAA 3′
p3b–EGFP and p3b–3×FLAG	Wild-type	Sense^*a*,*c*^ Antisense^*a*,*d*^	5′ TCTGCA**GAATTC** GCCGCCACCATGCCAAACTTCAGCT 3′ 5′ CCCCTT**GGATCC**TTTTCGCGCTGCGTTTAGAA 3′
p3b–EGFP	N-terminal deletions	Antisense^*e*^	5′ CATGGTGGCGGCGAATTC 3′
	D2–15 D2–25 D2–35 D2–45 D2–50 D2–55 D2–65	Sense	5′ CTGTTTAATATCACAGTTTACGATTTTTGTG 3′ 5′ GCTAAAAACTGGTATAAGTTACCTTTTG 3′ 5′ GTCAGATTACGTATCATAAATAATACAAAACC 3′ 5′ CCTAAAACAGCAAGTACTATAAAACG 3′ 5′ ACTATAAAACGTAGAAGAAGG 3′ 5′ AGAAGGGTTGTTGATTACAGAAAAATTG 3′ 5′ ATTCTAAACGCAGCGCGAAAAG 3′
p3b–EGFP	C-terminal deletions	Sense^*f*^	5′ GGATCCTTGGTGAGCAAG 3′
	D11–72 D21–72 D31–72 D41–72 D51–72 D56–72 D61–72	Antisense	5′ ACTCTTCAATATCCAGCTG 3′ 5′ TGTGATATTAAACAGTCGTATG 3′ 5′ ATACCAGTTTTTAGCACAAAAATC 3′ 5′ GATACGTAATCTGACTGC 3′ 5′ ACTTGCTGTTTTAGGTTTTG 3′ 5′ TCTACGTTTTATAGTACTTGC 3′ 5′ ATCAACAACCCTTCTTCTAC 3′
p3b–EGFP	Deletion of pat4 motifs (D53–57)	Sense Antisense	5′ GTTGTTGATTACAGAAAAATTG 3′ 5′ TATAGTACTTGCTGTTTTAG 3′

*a,* bold nucleotides are the restriction sites.

*b*, mutated 3b start codon (italic).

*c*, underlined nucleotides represent the Kozak sequence.

*d*, absent 3b stop codon.

*e*, common reverse primer for N-terminal truncations.

*f*, common forward primer for C-terminal truncations.

### Cell culture and transfection

Crandell Rees feline kidney (CRFK) cells were grown on coverslips in 24-well cell culture plates in minimum essential medium (MEM; Gibco BRL) supplemented with 5 % (v/v) foetal bovine serum (Sigma-Aldrich) and 2 % (w/v) lactalbumine (BD Biosciences) at 37 °C and 5 % CO_2_. Similarly, fIECs were grown in Dulbecco’s modified Eagle’s medium (DMEM; Gibco BRL)/Ham’s F12 Nutrient Mixture (Gibco BRL) (1 : 1, v/v) supplemented with 5 % (v/v) foetal bovine serum (Sigma-Aldrich) and 1 % (v/v) non-essential amino acids 100× (Gibco BRL). When reaching 60–80 % confluency, 24 h post-seeding, cells were transfected with 1 µg of plasmid DNA using Lipofectamine 2000 (Invitrogen). Six hours post-transfection, cells were washed and fresh medium with antibiotics (100 U ml^−1^ penicillin, 0.1 mg ml^−1^ streptomycin and 0.1 mg ml^−1^gentamycin) was added.

### Immunofluorescence

Twenty-four hours post-transfection, expression of the 3b protein was assessed by immunofluorescence. For the EGFP fusion constructs, cells were fixed with 4 % (w/v) paraformaldehyde (PF) in phosphate-buffered saline (PBS) for 10 min at room temperature (RT) and nuclei were stained with Hoechst 33 342 (Invitrogen) for 10 min at RT. The coverslips were washed twice with PBS, mounted in 0.9 : 0.1 (v/v) glycerin/PBS with 2.5 % (w/v) 1,4-diazabicyclo (2,2,2) octane and analysed by fluorescence microscopy (Leica Microsystems DMRBE). For the constructs containing the 3×FLAG-tag, cells were fixed with 4 % (w/v) PF in PBS for 10 min at RT, followed by permeabilization with 0.1 % (v/v) Triton X-100 for 2 min at RT. Next, coverslips were incubated with rabbit anti-FLAG antibody (Sigma-Aldrich) dilutions containing 10 % (v/v) negative goat serum for 1 h at 37 °C, followed by incubation with FITC-conjugated goat anti-rabbit antibodies (Invitrogen) for 1 h at 37 °C and Hoechst staining of the nuclei. To evaluate the dynamic localization of FCoV 3b, CRFK cells transfected with p3b–EGFP were fixed with PF at different time points (1, 2, 3, 4, 5, 6, 9, 12 and 18 h post-transfection) and nuclei were counterstained with Hoechst.

To assess mitochondrial localization, live cells were labelled with the MitoTracker Red CMXRos (100 nM, Cell Signaling Technology), followed by fixation and Hoechst staining of the nuclei. To determine colocalization with nucleoli, cells were fixed and permeabilized and subsequently incubated with rabbit anti-nucleolin antibody dilutions (Abcam) containing 10 % (v/v) negative rabbit serum. After two washes with PBS, Texas red-conjugated goat anti-rabbit IgG antibody dilutions (Invitrogen) were added for 1 h at 37 °C. Nuclei were stained with Hoechst and coverslips were washed twice with PBS, mounted and examined for fluorescence.

### Western blot analysis

Twenty-four hours post-transfection, cells were lysed for immunoblotting in radioimmunoprecipitation assay (RIPA) lysis buffer (Abcam) supplemented with the Halt Protease Inhibitor Cocktail (Thermo Scientific) for 1 h at 4 °C. After centrifugation, the supernatant was fractionated on a 12 % polyacrylamide gel by SDS-PAGE and then transferred to a Hybond-P PVDF membrane. After blotting, the membranes were blocked in 5 % (w/v) non-fat dry milk in PBS with 0.1 % (v/v) Tween-20 for 1 h at RT. The blots were further incubated overnight with ABfinity recombinant rabbit GFP monoclonal antibodies (Invitrogen) or rabbit anti-FLAG polyclonal antibodies (Sigma-Aldrich) at 4 °C. After being washed three times with Tris-buffered saline with 0.1 % (v/v) Tween-20, the blots were incubated for 1 h at RT with HRP-conjugated goat anti-rabbit antibodies (DakoCytomation). After three washing steps, proteins were visualized by enhanced chemiluminescence (ECL Prime, GE Healthcare) and analysed with the ChemiDoc MP Imaging System (Bio-Rad).

### Prediction and comparative sequence alignment

The FECV UCD 3b protein sequence was run through the PSORT II server (https://psort.hgc.jp) to predict the protein localization sites in cells. Potential nuclear export signals were identified using LocNES (http://prodata.swmed.edu/LocNES) and Wregex (http://wregex.ehubio.es). The MitoFates server (http://mitf.cbrc.jp) was used to assess the presence of mitochondrial localization sequences. A hydrophilicity plot of FCoV 3b protein based on the amino acid sequence analysis according to Hopp and Woods [63] was obtained with ProtScale from ExPASy (https://web.expasy.org/protscale/). To further identify conserved residues that may be involved in subcellular localization, the FECV UCD 3b amino acid sequence (GenBank accession number AMD11177.1) was compared with the FECV UG-FH8 3b sequence, kindly provided by Lars Larsen’s group (GenBank accession number ASU62490.1) and 176 other (putative) serotype I FCoV 3b amino acid sequences that were obtained from the National Center for Biotechnology Information (NCBI) Protein database (https://www.ncbi.nlm.nih.gov/protein) and provided by Bank-Wolf *et al*. [64], Brown *et al*. [65], Desmarets *et al*. [62], Hora *et al*. [66], Lewis *et al*. [67], Meszaros *et al*. [25] and Terada *et al*. [68]. The GenBank accession numbers can be found in Table S1 (available in the online version of this article). mega 7 software was used to perform multiple sequence alignment.

### Site-directed mutagenesis

Serial 3b truncated mutants were engineered by designing several forward and reverse primers that flank the region to be deleted (Table 1). The p3b–EGFP plasmid was amplified with these primers using Q5 DNA polymerase (New England Biolabs), followed by DpnI (New England Biolabs) and T4 polynucleotide kinase (New England Biolabs) treatment and ligation with the T4 DNA ligase (Promega). All plasmid sequences were confirmed by sequencing analysis. Transfection and immunofluorescent stainings were performed as described above.

## Results and discussion

### Expression of the FCoV 3b protein in transfected cells

**Fig. 1.**
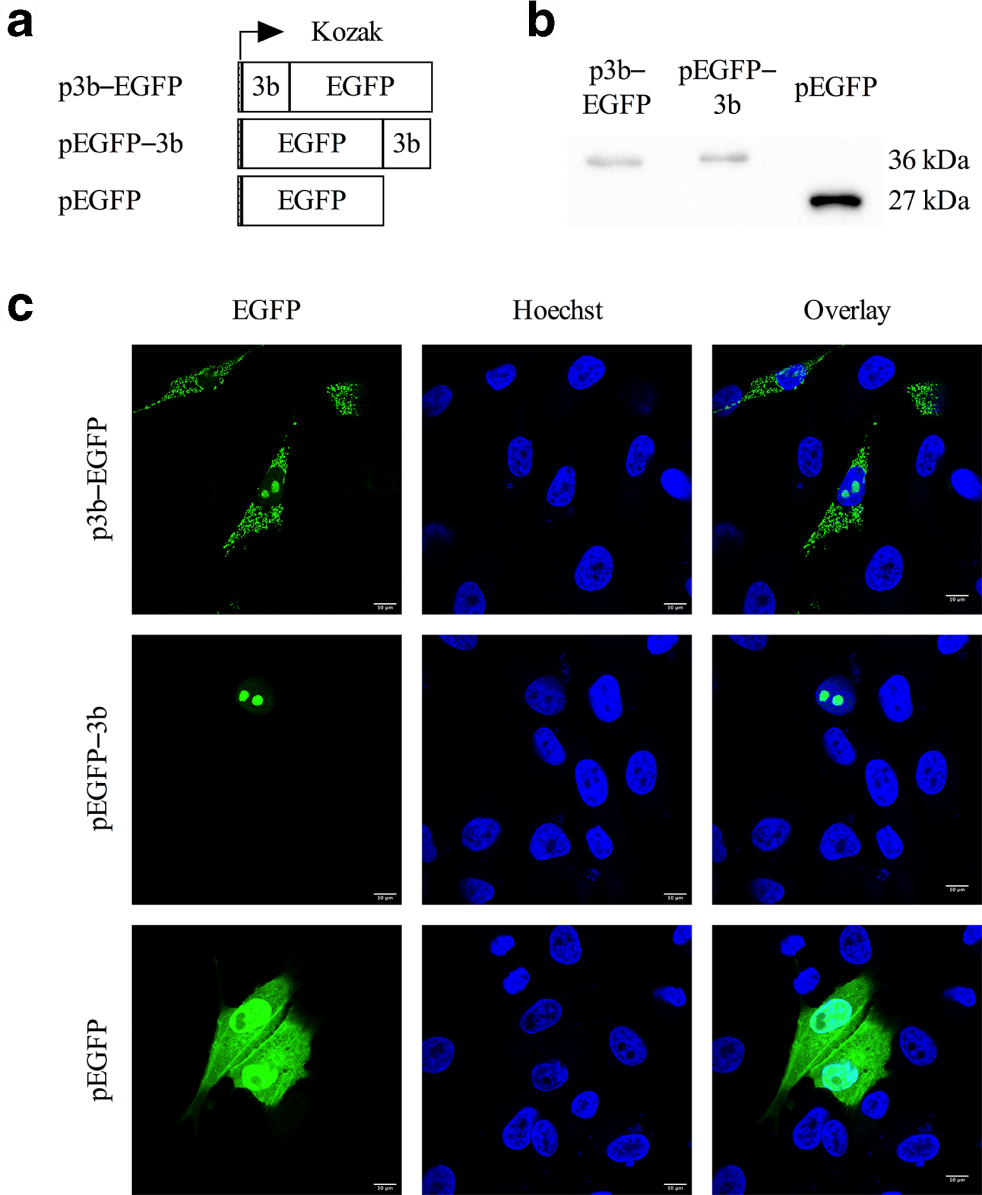
Expression of FCoV 3b protein in transfected CRFK cells. (a) Schematic overview of EGFP fusion constructs. (b) Western blot analysis. Twenty-four hours after transfection with p3b–EGFP, pEGFP–3b and pEGFP, cell lysates were prepared and fractioned by SDS-PAGE. After transfer, the PVDF membrane was probed with monoclonal anti-GFP antibodies. The molecular mass of the EGFP protein is ~27 kDa and after fusion with 3b, it is ~36 kDa. (c) Cellular localization of the EGFP constructs. CRFK cells were transfected with p3b–EGFP, pEGFP–3b and pEGFP. Cells were fixed 24 h post-transfection, the nuclei were stained with Hoechst (blue) and localization of the fusion protein (green) was assessed by confocal microscopy.

**Fig. 2.**
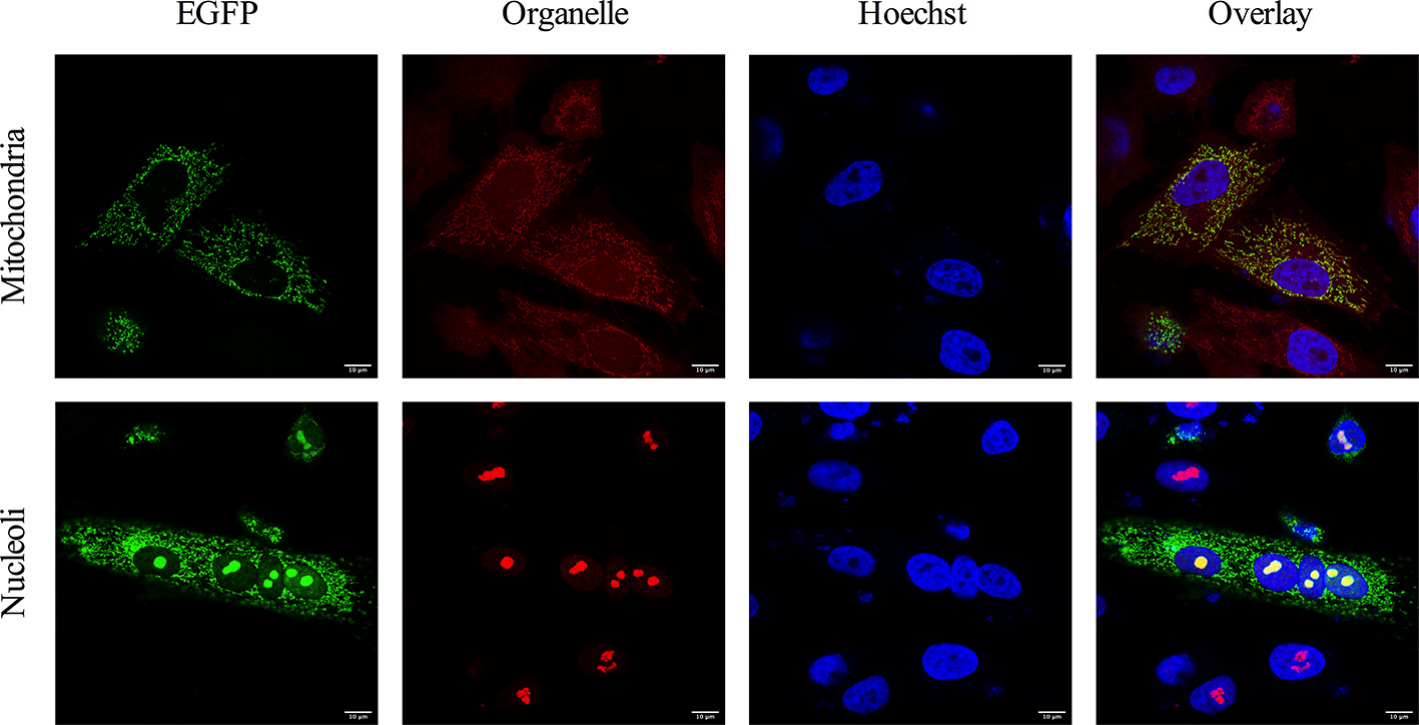
Colocalization of FCoV 3b protein with mitochondria and nucleoli. CRFK cells were transfected with p3b–EGFP (green) and colocalization of 3b with mitochondria and nucleoli (red) was evaluated 24 h after transfection. Mitochondria were visualized by labelling live cells with MitoTracker Red CMXRos, followed by fixation and Hoechst staining (blue) of the nuclei. To stain the nucleoli, cells were fixed and permeabilized, incubated with anti-nucleolin antibodies and Texas red-conjugated antibodies, and the nuclei were counterstained with Hoechst (blue).

Bioinformatics analysis using the PSORT II server predicted the 3b protein to localize to the cytoplasm (47.8 %), mitochondria (26.1 %), nucleus (21.7 %) or secretory vesicles (4.3 %). To examine its subcellular localization, the FCoV 3b coding information was cloned in pcDNA3.1(−)-EGFP, fusing it either at the C-terminus (p3b–EGFP) or at the N-terminus (pEGFP–3b) with EGFP (Fig. 1a). Next, CRFK cells were transfected with these constructs and the subcellular distribution pattern was evaluated by confocal microscopy (Fig. 1c). Wild-type EGFP, which functions as the control vector, showed a diffuse distribution throughout the cytoplasm and the nucleus, but was excluded from the nucleoli, as described by other authors (i.e. in [69, 70]). Transfection of the p3b–EGFP expression vector resulted in a dotted or tubular distribution in the cytoplasm. Visualization of the mitochondria using MitoTracker Red CMXRos clearly demonstrated that FCoV 3b–EGFP colocalizes with this organelle (Fig. 2). As mitochondria are dynamic structures characterized by fusion and fission, they can occur either as individual structures or form a large interconnected tubular network [49], which could explain the fact that some cells show a more dotted cytoplasmic 3b expression pattern and others a more tubular one.

Although the majority of cells exclusively expressed the 3b–EGFP fusion protein in mitochondria, accumulation in certain regions of the nucleus was apparent in some cells (Fig. 1c). These subnuclear compartments corresponded with nucleoli, as confirmed by immunofluorescent staining (Fig. 2). Western blot analysis using anti-GFP antibodies showed the migration of chimeric proteins at the expected molecular mass of approximately 36 kDa (Fig. 1b). These results are in line with the observations from Meszaros and colleagues, who investigated the subcellular localization of all FCoV ORF3-encoded proteins [25]. Interestingly, fusion of 3b at the C-terminus of EGFP abolished mitochondrial translocation and 3b expression was restricted to the nucleoli (Fig. 1c), which may indicate the presence of an important N-terminal targeting signal to allow mitochondrial localization.

**Fig. 3.**
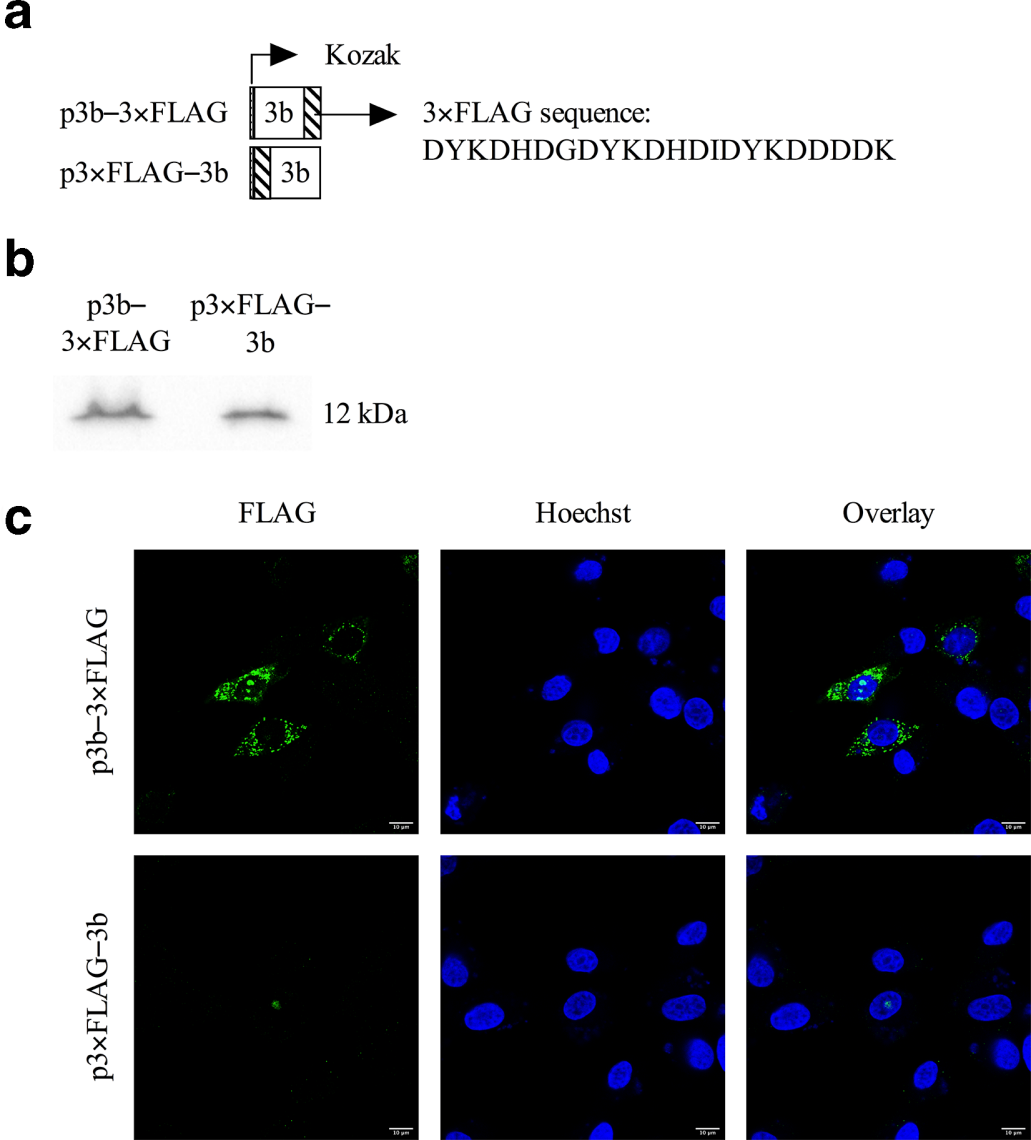
Expression of FCoV 3b protein in CRFK cells transfected with 3×FLAG constructs. (a) Schematic overview of 3×FLAG fusion constructs. (b) Western blot analysis. Twenty-four hours after transfection with p3b–3×FLAG and p3×FLAG–3b, cell lysates were prepared and fractioned by SDS-PAGE. After blotting, the PVDF membrane was probed with polyclonal anti-FLAG antibodies. The expected molecular mass of p3b–3×FLAG and p3×FLAG–3b is ~12 kDa. (c) Cellular localization of the 3×FLAG constructs. CRFK cells were transfected with p3b–3×FLAG and p3×FLAG–3b. Twenty-four hours post-transfection, cells were fixed, permeabilized and incubated with rabbit anti-FLAG antibodies, followed by incubation with FITC-conjugated goat anti-rabbit antibodies. The nuclei were stained with Hoechst (blue) and localization of the fusion protein (green) was assessed by confocal microscopy.

**Fig. 4.**
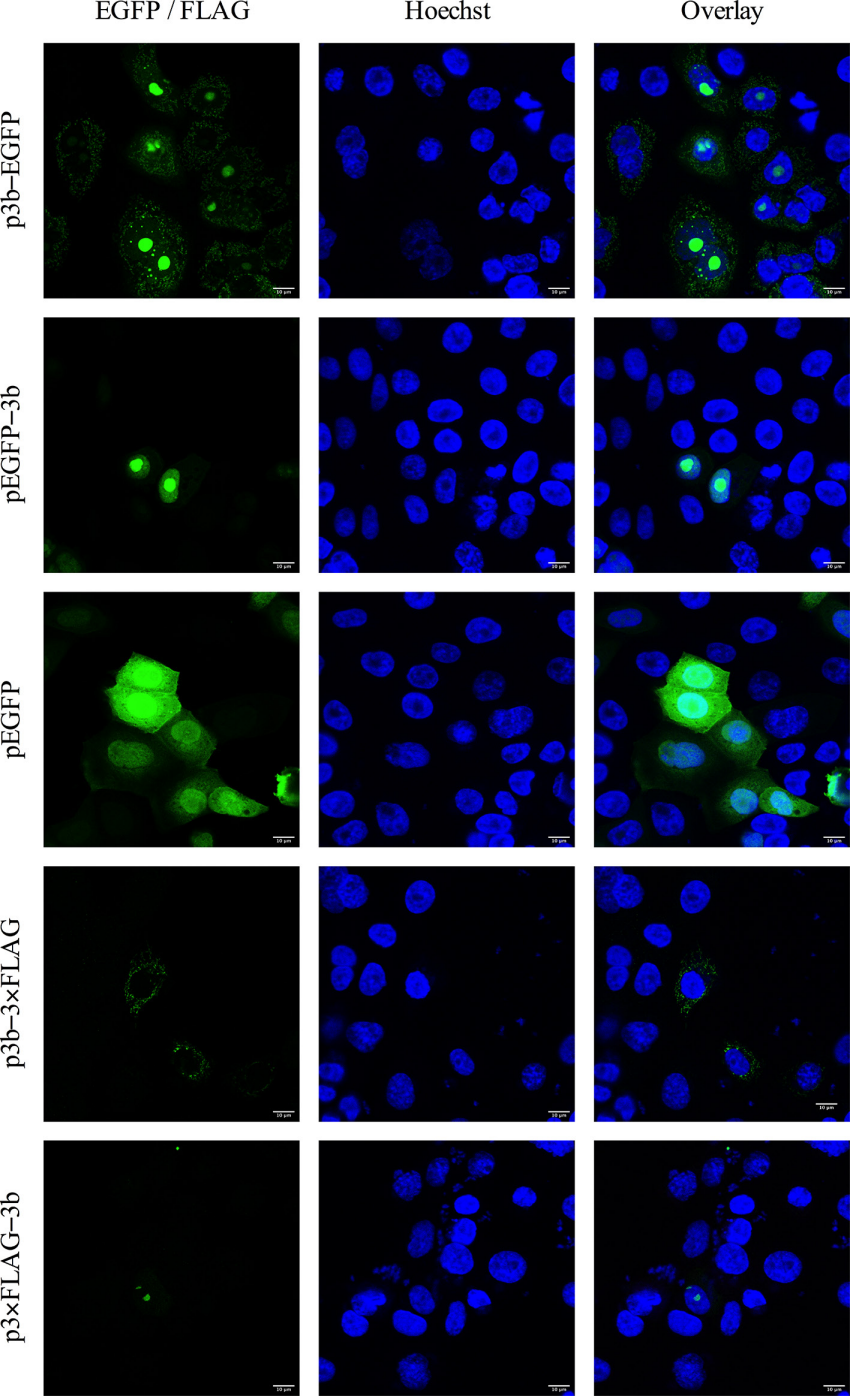
Expression of FCoV 3b protein in transfected fIECs. Feline intestinal epithelial cells were transfected with the EGFP and 3×FLAG fusion constructs. Cells were fixed 24 h post-transfection and probed with anti-FLAG antibodies for the 3×FLAG constructs. The nuclei were stained with Hoechst (blue) and localization of the fusion protein (green) was assessed by confocal microscopy.

Due to the lack of specific anti-3b antibodies and to exclude a potential effect of EGFP on subcellular localization, 3b was also fused with a 3×FLAG-tag either N-terminally or C-terminally (p3×FLAG–3b and p3b–3×FLAG, respectively) (Fig. 3a) and expression of the tagged 3b proteins was confirmed by Western blot analysis, revealing bands with the expected molecular weight of approximately 12 kDa (Fig. 3b). Similar results were obtained as for the EGFP fusion proteins, i.e. mitochondrial and nucleolar expression for p3b–3×FLAG and only nucleolar expression for p3×FLAG–3b (Fig. 3c). The fluorescent signal was, however, weaker compared to EGFP. In addition, these EGFP and 3×FLAG constructs were also introduced in fIECs to exclude cell type-dependent localization of 3b. These fIECs are in fact the target cells for FECV and to date the only cell line in which serotype I strains can be propagated *in vitro* [19]. Even though the transfection efficiency is quite low in this epithelial cell line, the 3b expression pattern was comparable to that observed in CRFK cells (Fig. 4). From these results, we can conclude that the 3b localization is not influenced by the fusion protein or tag to which it was fused nor by the cell type in which the protein is expressed, and thus most likely represents the actual subcellular localization of FCoV 3b. Furthermore, it was shown by Meszaros and colleagues that the 3b expression pattern did not change when transfection was followed by infection with FIPV DF-2, although 3b accumulation in the nucleolus was more frequently observed in infected cells compared to transfection only [25].

### Determination of the peptide domains involved in nucleolar localization

Proteins that target the nucleolus first migrate to the nucleus and then subsequently to the nucleolus. The active transportation of proteins through the nuclear pore complex is usually driven by NLSs. Classical NLSs include the monopartite pat4 and pat7 motifs and the bipartite motif (reviewed in [38, 40, 41, 71]). The pat4 motif is a continuous stretch of four basic amino acid residues (arginine or lysine) [β_4_] or three basic amino acid residues followed by a histidine or proline [β_3_(H/P)]. The pat7 motif starts with a proline and is followed, within three residues, by a basic four-residue cluster containing at least three arginines or lysines [Pχχ(β_3_χ)]. Bipartite NLSs have 2 clusters of basic amino acids separated by a 10–12 amino acid-long spacer, where the first cluster consists of 2 basic residues and the second cluster is a basic 5-residue region consisting of at least 3 basic residues [ββχ_(10-12)_(β_3_χ_2_)] [72, 73]. NLS diversity keeps expanding as non-classical NLSs (reviewed in [38]) and additional NLS classes have been identified [74]. It should be noted that, in the absence of an NLS, proteins can still localize to the nucleus by co-transportation with other proteins that contain an NLS [75, 76]. In addition, the presence of an NLS does not guarantee nuclear localization, as the NLS may be masked from the protein surface (reviewed in [42]). NoLSs that drive nucleolar targeting are poorly characterized and usually closely associated with NLSs, thereby hampering the identification of a consensus motif. It is important, however, to differentiate between (1) NLS-only signals, which will target proteins to the nucleus but not to nucleoli, (2) NoLS-only signals, which will translocate proteins to the nucleoli, but are not capable of transporting them through the nuclear pore complex, and (3) joint NoLS–NLS signals, which will target the proteins to the nucleus and subsequently induce nucleolar accumulation [45].

**Fig. 5.**
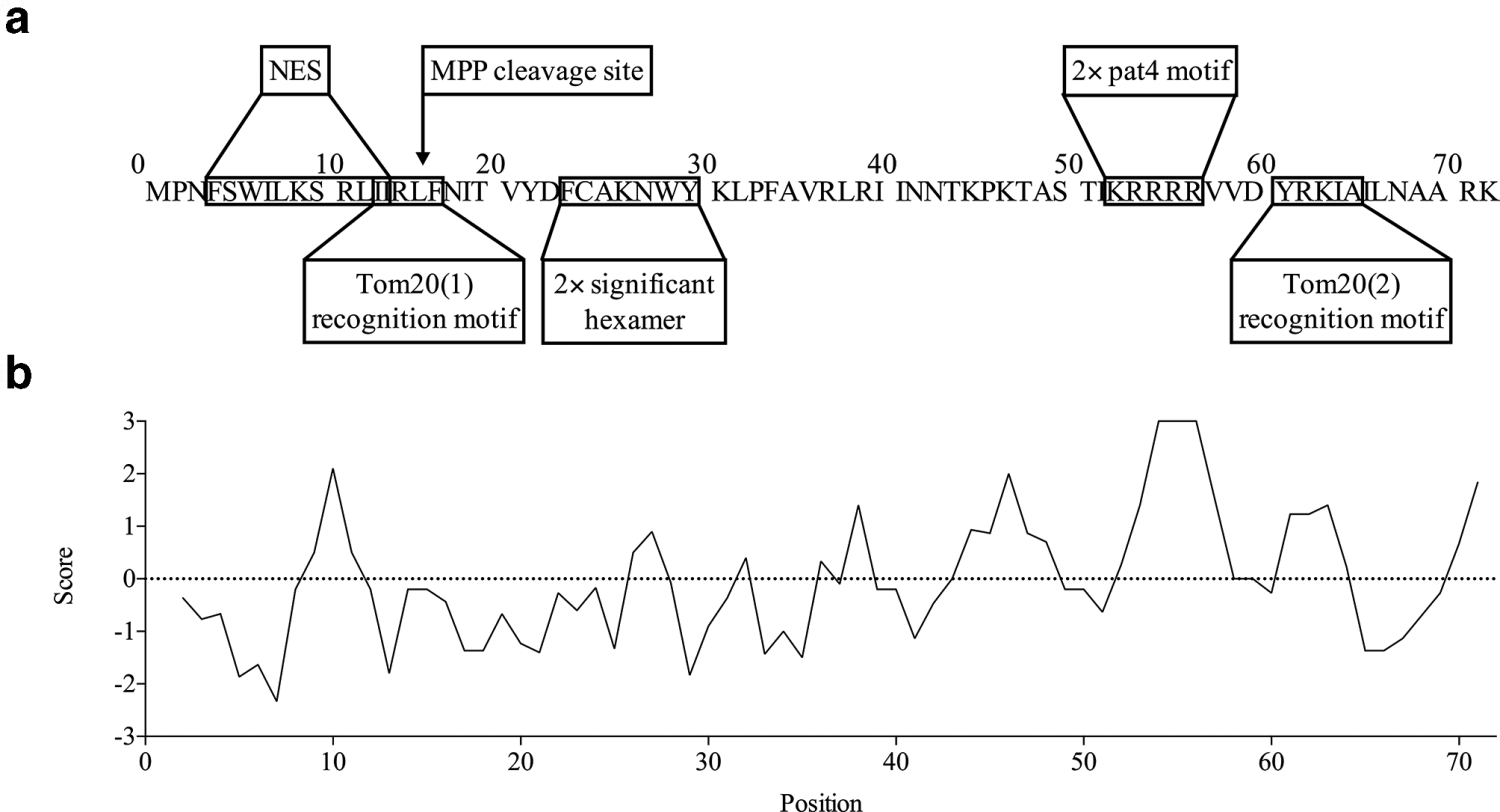
Predicted peptide domains involved in subcellular localization of 3b. (a) Schematic overview of the predicted molecular determinants involved in nuclear and mitochondrial localization of 3b. The PSORT II server identified two overlapping pat4 motifs that may be involved in nuclear translocation. A potential NES was predicted by the LocNES and Wregex. MitoFates located two putative Tom20 recognition motifs, which may be involved in binding of 3b to Tom20 in the outer membrane of the mitochondria. Additionally, a potential MPP cleavage site and two overlapping significant hexamers were observed. (b) Hopp−Woods hydrophilicity plot of FCoV 3b protein.

A potential NLS is present in the FCoV 3b amino acid sequence, as the PSORT II server identified two putative (overlapping) pat4 motifs (amino acids 53 to 56 and 54 to 57) (Fig. 5a). In addition, a Hopp–Woods hydrophilicity plot (Fig. 5b) shows that these overlapping pat4 motifs are located in a hydrophilic region and therefore the surface should be easily accessible for interaction with importins. To investigate if these residues are conserved amongst different strains, 178 (putative) serotype I FCoV 3b protein sequences were compared. All of them contained at least one pat4 motif in the same region. Thirteen (7.30 %) had a single pat4 motif and 165 (92.7 %) had 2 overlapping pat4 motifs. There is a 90 % amino acid homology between the 3b sequence described by Meszaros and colleagues (isolate HU2009FC, GenBank accession number AWU66525.1), which was also shown to localize to nucleoli, and the FECV UCD 3b strain used in this study. Both isolates have two overlapping pat4 motifs, with a slightly different amino acid sequence (KRRKR and KRRRR, respectively). The nucleocapsid proteins of several members of the order *Nidovirales*, such as porcine reproductive and respiratory syndrome virus (PRRSV, family *Arteriviridae*) [70, 77–80], transmissible gastroenteritis virus (TGEV) [81], mouse hepatitis virus (MHV) [81, 82] and infectious bronchitis virus (IBV) [82–85] (family *Coronaviridae*), also target the nucleolus and have been shown to incorporate one or more potential NLSs. Similarly, the SARS-CoV 3b protein was predicted to have two overlapping NLSs, a pat4 and bipartite motif, that were shown to be associated with nuclear (nucleolar) localization [27].

**Fig. 6.**
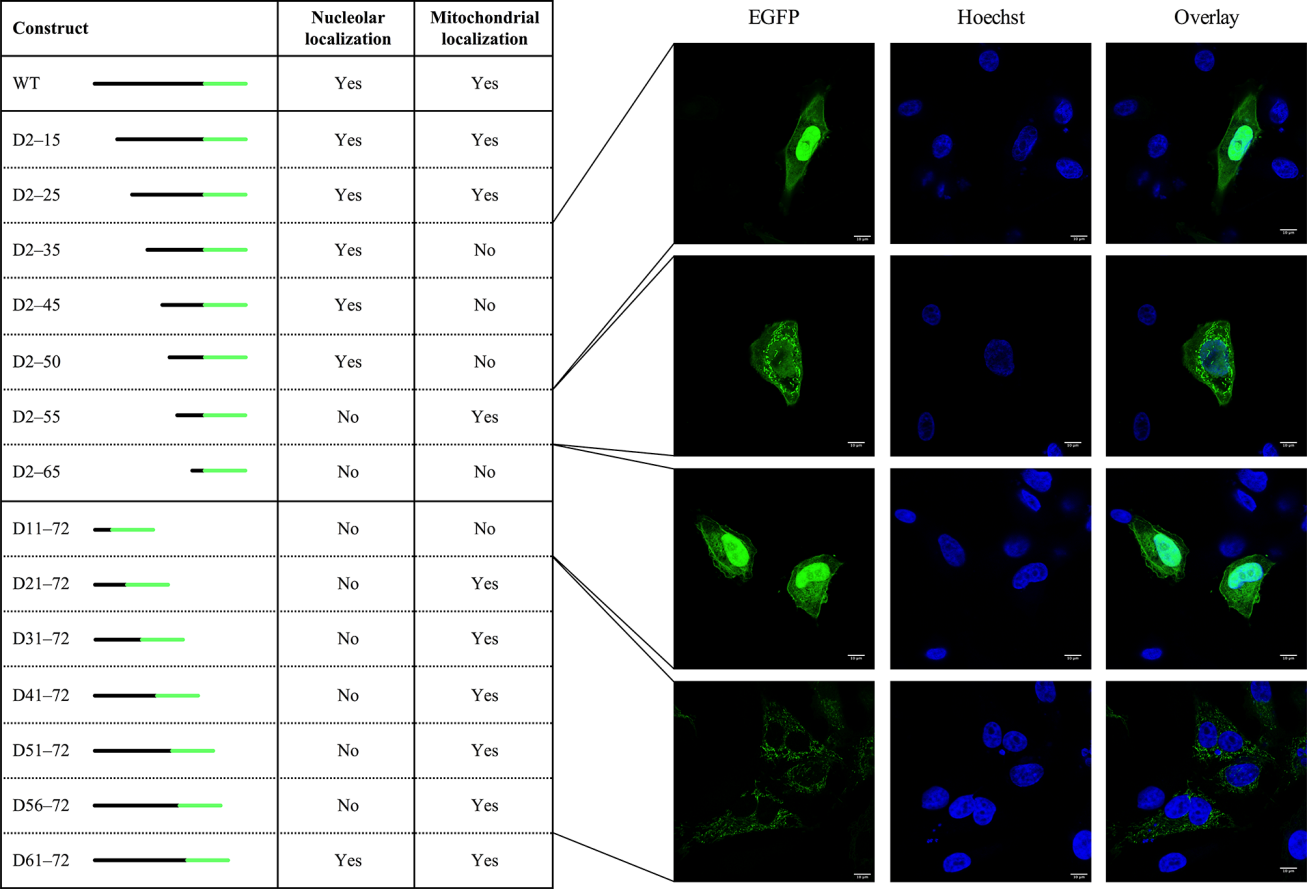
Subcellular localization of 3b truncated mutants. N-terminal and C-terminal mutants of p3b–EGFP were constructed by site-directed mutagenesis. Next to the name of the truncated constructs, a schematic representation is given where the black line represents the truncated 3b part of the protein sequence and the green line represents EGFP. For each of the truncated constructs, nucleolar and mitochondrial translocation was evaluated after transfection of CRFK cells. The images on the right show the localization pattern of EGFP for the respective truncations.

To examine which peptide domains are responsible for the observed nucleolar expression pattern of FCoV 3b, a set of consecutive truncations, starting from both the N-terminus and the C-terminus of the 3b protein, were constructed and expressed in CRFK cells. As shown in Fig. 6, deletion of residues 2 to 50 (p3b–EGFP D2–15, D2–25, D2–35, D2–45 and D2–50) still resulted in EGFP translocation to the nucleoli. However, deletion of residues 2 to 55 (p3b–EGFP D2–55), which results in a deletion of the first three residues of the pat4 motifs, and deletion of residues 2 to 65 (p3b–EGFP D2–65), whereby the complete pat4 motifs are removed, was associated with a complete loss of nucleolar accumulation of the corresponding EGFP fusion protein. Similarly, deleting the 12 C-terminal residues (p3b–EGFP D61–72) did not alter nucleolar localization, but deletion of the 17 C-terminal residues (p3b–EGFP D56–72), which results in a deletion of the last 2 residues of the pat4 motifs, or deletion of the 22 C-terminal residues (p3b–EGFP D56–72), whereby both pat4 motifs are completely removed, abrogated nucleolar expression. In addition, deletion of the overlapping pat4 motifs (p3b–EGFP D53–57), also resulted in a lack of nucleolar localization. These results suggest that, even though the 3b protein is theoretically able to passively diffuse through the nuclear pore complex, translocation to the nucleus is likely to be an active process driven by a joint NoLS–NLS signal.

### Identification of the molecular determinants involved in mitochondrial translocation

Mitochondrial localization is often driven by the presence of an mTP. As mTPs have no consensus motif, prediction is frequently based on (1) the amino acid composition of the N-terminal part of the protein, (2) the propensity to form amphipathic α-helices and (3) the presence of putative recognition motifs or MPP cleavage sites. A conserved motif ϕχχϕϕ in the N-terminal mitochondrial presequence, where ϕ represents hydrophobic residues (L, F, I, V, W, Y, M) and χ any residue, that is recognized by the Tom20 protein has been identified [86, 87], with a preference for basic residues on position 3 (ϕχβϕϕ, with β representing either R, K or H) [87]. However, import into mitochondria can still be observed after substituting such hydrophobic residues with alanine, despite reduced Tom20 binding [88]. In addition, 14 hexamers, such as ϕϕσβϕϕ, ϕϕβσϕϕ and βϕϕσσσ (where σ represents polar residues S, T, N or Q), have been proposed to be involved in mitochondrial targeting, but their actual function as mitochondrial targeting sequences remains to be elucidated [89]. In the absence of an N-terminal mTP, proteins can still target the mitochondria through internal targeting signals or hydrophobic domains (reviewed in [51]). Mitochondrial targeting of the SARS-CoV 3b protein was shown to be determined by a region located in the middle of the protein rather than by N-terminal residues [26, 28]. Furthermore, the outer membrane of the mitochondria contains proteins, called porins, that will freely allow molecules smaller than 10 kDa to cross the mitochondrial membrane [46]. In addition, viral proteins can also be transported from the ER into the mitochondria via the mitochondria-associated membrane compartment (reviewed in [51, 90]).

The N-terminal part of the FCoV 3b protein mainly consists of hydrophobic and positively charged residues, whereas negatively charged residues are nearly absent. MitoFates allocated the maximum positively charged amphiphilicity score to residues 10 to 19. Furthermore, MitoFates identified two putative Tom20 recognition motifs [amino acids 13 to 17 and 61 to 65, further annotated as Tom20(1) and Tom20(2), respectively] with a consensus ϕχβϕϕ pattern (Fig. 5a). Multiple sequence alignment revealed that 173 (97.19 %) and 176 (98.88 %) of a total of 178 aligned sequences respectively have a Tom20(1) and Tom20(2) recognition motif. The amino acid composition varied, however, amongst different strains: I/L-I/L/V-R-L-F/Y for Tom20(1) and Y-R/G-K/R-I/V-A for Tom20(2). Five isolates (2.81 %) seemed to lack a Tom20(1) motif and two isolates (1.12 %) a Tom20(2) motif (Table 2). Comparison of the 3b sequence of isolate HU2009FC, which is known to locate to mitochondria [25], and FECV UCD showed that the Tom20(1) and Tom20(2) recognition motifs were identical. In addition to these Tom20 recognition motifs, two overlapping hexamers (amino acids 24 to 29 and 25 to 30) with a ϕϕϕβσϕ and ϕϕβσϕϕ pattern, respectively, and potentially associated with mitochondrial targeting, were identified by MitoFates. Of the 178 aligned 3b sequences, 155 (87.08 %) had at least 1 hexamer in this region. There was, however, great variability between isolates in terms of amino acid composition. Finally, an MPP cleavage site between residue 16 and 17 was proposed (Fig. 5a). MPP cleavage sites are often characterized by the presence of arginine at position –2, –3 or −10 relative to the cleavage site (reviewed in [91]).

**Table 2.** FCoV serotype I strains with deviant Tom20 recognition motifs

Strain/isolate	GenBank accession no.	Tom20(1) motif	Tom20(2) motif
4662inCJB07	ACI13613	Yes	No (YRGIA)
FIPV12F	AIL54196	No (IIGVC)	Yes
FIPV04A	AIL54201	No (IIQLF)	Yes
FIPV09A	AIL54208	No (IIQLF)	Yes
FIPV09F	AIL54210	No (IIQLF)	Yes
USP7/faeces/FIP+/local3/C27	AIN55828	Yes	No (YRRRRIA)
USP7/faeces/FIP+/local3/C17	AIN55946	No (II---)	Yes

As for nucleolar localization, mitochondrial targeting was assessed after the transfection of different truncated 3b mutants fused to EGFP (Fig. 6). The constructs with C-terminal deletions of residues 21 to 72 (p3b–EGFP D21–72, D31–72, D41–72, D51–72, D56–72 and D61–72) all continued to target EGFP to the mitochondria, but p3b–EGFP D11–72 was distributed diffusely throughout the cytoplasm and a mitochondrial expression pattern was no longer observed. For the N-terminal truncated mutants, deletion of residues 2 to 25 (p3b–EGFP D2–15 an D2–25) did not seem to influence mitochondrial colocalization, but mitochondrial expression was abrogated when amino acids 2 to 35 (p3b–EGFP D2–35) were deleted. These results suggest the presence of an N-terminal mTP with a critical role for residues 10 to 35, which comprise both the predicted Tom20(1) motif and the identified hexamers, in mitochondrial localization. However, it remains unclear what specific role is exerted by the Tom20(1) motif and the identified hexamers, as deletion of one of them has no effect on mitochondrial expression, but deletion of both constrains localization to the mitochondria.

Interestingly, after transfection of p3b–EGFP D2–55, the fluorescent signal was diffusely present in the cytoplasm as for p3b–EGFP D2–35, D2–45 and D2–50, but additionally the typical mitochondrial expression pattern could also be observed (Fig. 6). This could be a consequence of the presence of the Tom20(2) motif that is now in the N-terminus of this truncated protein. Furthermore, deletion of residues 2 to 65 (p3b–EGFP D2–65), which includes deletion of the Tom20(2) motif, no longer induced mitochondrial colocalization. It should be noted that as a diffuse cytoplasmic pattern was observed after the transfection of p3b–EGFP D2–55, in addition to mitochondrial expression, there may be an indication that mitochondrial localization of this truncated protein is reduced compared to the wild-type 3b. This could be explained by the presence of an alanine at position 5 of the Tom20(2) motif YRKIA, as Mukhopadhyay and colleagues have related that alanine substitution of the hydrophobic residues is associated with reduced Tom20 binding [88].

### Dynamic localization of FCoV 3b

**Fig. 7.**
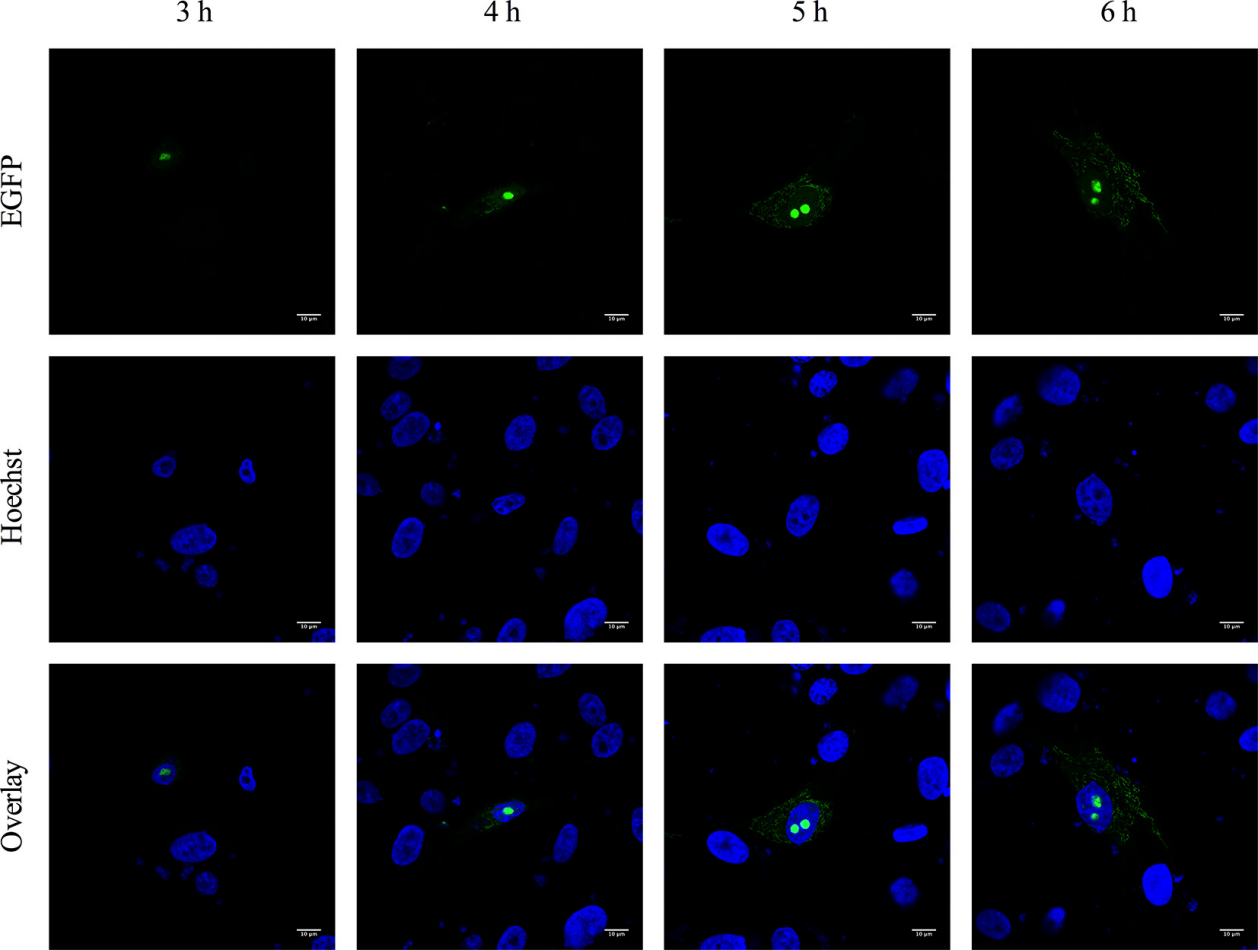
Dynamic localization of FCoV 3b. Confocal microscopy of CRFK cells expressing 3b–EGFP (green) at 3, 4, 5 and 6 h post-transfection. Nuclei were stained with Hoechst (blue).

To assess the dynamics of 3b expression and the potential trafficking of 3b from the nucleus to the cytoplasm or vice versa, 3b translocation was assessed at different time points post-transfection. Faint expression in the nucleoli was observed 3 h post-transfection (Fig. 7). At 4 h post-transfection, the fluorescent signal in the nucleoli was found to increase and a vague cytoplasmic signal was detected, but it was not until 5 h after transfection that a mitochondrial pattern was clearly visible. As of 6 h post-transfection, the fluorescent signal was brighter and an increasing number of cells began to show exclusive mitochondrial expression of the 3b protein, as exemplified in Fig. 2. These results may indicate nucleolar–mitochondrial shuttling, as observed for the PRRSV nucleocapsid [78]. Like nuclear import, shuttling proteins from the nucleus back to the cytoplasm is established by a nuclear export signal (NES) that is recognized by exportins such as CRM1. A well-characterized NES is the export motifϕχ_(2-3)_ϕχ_(2-3)_ϕχϕ (where ϕ represents one of the hydrophobic residues L, V, I, F or M and χ is any residue) [92, 93], but this consensus pattern has been progressively expanded and computational methods have been addressed to further model the sequence diversity of NESs [93–100].

A potential NES was identified in the FCoV 3b sequence – FSWILKSRLII (amino acids 4 to 14) – by both the LocNES and Wregex prediction tools (Fig. 5a). However, despite the presence of this potential NES, the expression of the corresponding truncated 3b variants showed no indication of its actual functionality. Indeed, deletion of the first 25 amino acids (p3b–EGFP D2–15 an D2–25), including this potential NES, did not abrogate mitochondrial expression (Fig. 6). These results suggest that 3b may not migrate first to the nucleolus and subsequently to the mitochondria, or that another unidentified NES is present in the 3b sequence. An alternative hypothesis to account for the observed spatiotemporal kinetics is that nucleolar accumulation is much more concentrated compared to mitochondrial expression, thus facilitating the observation of fluorescent signal in the nucleoli. Furthermore, it was described that proteins that target both the nucleus and cytoplasmic structures usually contain multiple signals to target them to their subcellular compartments [32, 34, 39] and it was suggested by Garcia-Bustos and colleagues that the most N-terminal-located signal dominates [39]. As the putative mTP of the FCoV 3b is located more N-terminally compared to the NoLS, this may explain why many cells exclusively show mitochondrial expression.

### Concluding remarks

In the present study, we present data demonstrating that the FCoV 3b protein directs the localization of a fusion protein (EGFP) or tag (3×FLAG tag) to mitochondria and nucleoli, independently of the type of fusion protein and transfected cell type. Nucleolar localization requires a joint NoLS–NLS signal that encompasses two overlapping pat4 motifs. Moreover, the presence of a pat4 motif is highly conserved among serotype I FCoV strains. Mitochondrial expression is mainly triggered by N-terminal residues 10–35, which contain a putative Tom20 recognition motif and two overlapping hexamers potentially involved in mitochondrial localization. Additionally, a second Tom20 recognition motif was identified, which seems to be able to target the protein to mitochondria, although less efficiently compared to the N-terminal mTP. Our observations provide new insights into the intracellular trafficking of FCoV 3b and further research should focus on the specific functions 3b exerts in its target organelles to unravel the mechanisms that allow 3b to contribute to FCoV pathogenesis.

## Supplementary Data

Supplementary File 1
