# Supplementary material for: Identification of peptide domains involved in the subcellular localization of the feline coronavirus 3b protein
Source: J Gen Virol. 2019 Sep 4;100(10):1417–30. doi: 10.1099/jgv.0.001321 (PMC7079696; doi:10.1099/jgv.0.001321)
Supplement: Supplementary File 1 [file jgv-100-1417-s001.pdf]

Table S1. GenBank accession numbers used for multiple sequence alignment.

ACI13494.1	AIL54148.1	AIN55806.1	AIN55969.1
ACI13497.1	AIL54151.1	AIN55808.1	AIN55972.1
ACI13501.1	AIL54154.1	AIN55810.1	AIN55975.1
ACI13505.1	AIL54157.1	AIN55812.1	AIN55978.1
ACI13508.1	AIL54160.1	AIN55814.1	AIN55981.1
ACI13512.1	AIL54163.1	AIN55816.1	AIN55984.1
ACI13516.1	AIL54166.1	AIN55818.1	AIN55987.1
ACI13520.1	AIL54169.1	AIN55820.1	AIN55990.1
ACI13524.1	AIL54172.1	AIN55822.1	AIN55992.1
ACI13528.1	AIL54175.1	AIN55824.1	AIN55995.1
ACI13532.1	AIL54178.1	AIN55826.1	AIN55997.1
ACI13536.1	AIL54181.1	AIN55828.1	AIN55999.1
ACI13540.1	AIL54184.1	AIN55831.1	AIN56001.1
ACI13544.1	AIL54187.1	AIN55833.1	AIN56003.1
ACI13548.1	AIL54190.1	AIN55835.1	AIN56004.1
ACI13552.1	AIL54193.1	AIN55837.1	AJO26975.1
ACI13556.1	AIL54196.1	AIN55839.1	AJO26985.1
ACI13559.1	AIL54199.1	AIN55840.1	AJO26995.1
ACI13564.1	AIL54201.1	AIN55842.1	AJO27005.1
ACI13568.1	AIL54204.1	AIN55847.1	AJO27015.1
ACI13571.1	AIL54206.1	AIN55851.1	AJO27025.1
ACI13576.1	AIL54208.1	AIN55855.1	AMD11132.1
ACI13580.1	AIL54210.1	AIN55862.1	AMD11144.1
ACI13584.1	AIL54211.1	AIN55864.1	AMD11155.1
ACI13588.1	AIL54212.1	AIN55868.1	AMD11166.1
ACI13592.1	AIL54214.1	AIN55872.1	AMD11177.1
ACI13595.1	AIL54216.1	AIN55878.1	AMD11188.1
ACI13598.1	AIL54219.1	AIN55880.1	AMD11199.1
ACI13606.1	AIL54222.1	AIN55884.1	AMD11210.1
ACI13609.1	AIL54224.1	AIN55888.1	AMD11221.1
ACI13613.1	AIL54226.1	AIN55891.1	AMD11232.1
ACI13617.1	AIL54229.1	AIN55897.1	ASU62490.1
ACI13620.1	AIL54231.1	AIN55901.1	AWU66525.1
ACI13623.1	AIL54234.1	AIN55905.1	BAJ08257.1
ACI13627.1	AIL54237.1	AIN55909.1	
ACI13631.1	AIL54240.1	AIN55912.1	
ACI13635.1	AIL54242.1	AIN55931.1	
ACI13639.1	AIN55751.1	AIN55934.1	
ACI13643.1	AIN55755.1	AIN55937.1	
AIL54121.1	AIN55759.1	AIN55940.1	
AIL54124.1	AIN55764.1	AIN55943.1	
AIL54127.1	AIN55766.1	AIN55946.1	
AIL54130.1	AIN55769.1	AIN55951.1	
AIL54133.1	AIN55774.1	AIN55954.1	
AIL54136.1	AIN55778.1	AIN55957.1	
AIL54139.1	AIN55800.1	AIN55960.1	
AIL54142.1	AIN55802.1	AIN55963.1	
AIL54145.1	AIN55804.1	AIN55966.1	
